# “Genotype-first” approaches on a curious case of idiopathic progressive cognitive decline

**DOI:** 10.1186/s12920-014-0066-9

**Published:** 2014-12-03

**Authors:** Lingling Shi, Bingxiao Li, Yonglan Huang, Xueying Ling, Tianyun Liu, Gholson J Lyon, Anding Xu, Kai Wang

**Affiliations:** Guangdong-Hongkong-Macau Institute of CNS Regeneration, Jinan University, Guangzhou, Guangdong 510623 China; Guangdong Medical Key Laboratory of Brain Function and Diseases, Jinan University, Guangzhou, Guangdong 510623 China; GHM Collaboration and Innovation Center for Tissue Regeneration and Repair, Jinan University, Guangzhou, Guangdong 510623 China; Neonatal Intensive Care Unit, The 1st Affiliated Hospital, Jinan University, Guangzhou, Guangdong 510623 China; Department of Endocrinology and Metabolism, Guangzhou Women and Children’s Medical Center, Guangzhou, Guangdong 510623 China; Medical Imaging Center, The 1st Affiliated Hospital, Jinan University, Guangzhou, Guangdong 510623 China; Department of Genetics, Stanford University, Stanford, CA 94305 USA; Stanley Institute for Cognitive Genomics, Cold Spring Harbor Laboratory, Cold Spring Harbor, NY, 11797 USA; Department of Neurology, The 1st Affiliated Hospital, Jinan University, Guangzhou, Guangdong 510632 China; Zilkha Neurogenetic Institute, University of Southern California, Los Angeles, CA 90089 USA; Department of Psychiatry & Behavioral Sciences, University of Southern California, Los Angeles, CA 90089 USA

## Abstract

**Background:**

In developing countries, many cases with rare neurological diseases remain undiagnosed due to limited diagnostic experience. We encountered a case in China where two siblings both began to develop idiopathic progressive cognitive decline starting from age six, and were suspected to have an undiagnosed neurological disease.

**Methods:**

Initial clinical assessments included review of medical history, comprehensive physical examination, genetic testing for metabolic diseases, blood tests and brain imaging. We performed exome sequencing with Agilent SureSelect exon capture and Illumina HiSeq2000 platform, followed by variant annotation and selection of rare, shared mutations that fit a recessive model of inheritance. To assess functional impacts of candidate variants, we performed extensive biochemical tests in blood and urine, and examined their possible roles by protein structure modeling.

**Results:**

Exome sequencing identified *NAGLU* as the most likely candidate gene with compound heterozygous mutations (chr17:40695717C > T and chr17:40693129A > G in hg19 coordinate), which were documented to be pathogenic. Sanger sequencing confirmed the recessive patterns of inheritance, leading to a genetic diagnosis of Sanfilippo syndrome (mucopolysaccharidosis IIIB). Biochemical tests confirmed the complete loss of activity of alpha-N-acetylglucosaminidase (encoded by *NAGLU*) in blood, as well as significantly elevated dermatan sulfate and heparan sulfate in urine. Structure modeling revealed the mechanism on how the two variants affect protein structural stability.

**Conclusions:**

Successful diagnosis of a rare genetic disorder with an atypical phenotypic presentation confirmed that such “genotype-first” approaches can particularly succeed in areas of the world with insufficient medical genetics expertise and with cost-prohibitive in-depth phenotyping.

## Background

Next-generation sequencing (NGS) technologies have advanced many aspects of genomic sciences, and have accelerated the pace of discovery in biomedical research [1-3]. For example, NGS has been widely used in population genetics studies [4,5], metagenomics [6,7], agrigenomics [8,9], epigenetics [10] and gene expression studies [11,12]. In particular, human genome and exome sequencing can now be used in dissecting the genetic basis of diseases and traits that have proven to be intractable to conventional gene-discovery strategies [13-16]. In addition, NGS empowers clinical diagnostics and other aspects of medical practice, such as genetic diagnosis, disease prognosis, therapeutic target identification, optimization of treatment regimens, prenatal testing and personalized disease-risk profiling.

Besides identifying new mutations in genes previously implicated in known diseases, genome/exome sequencing has also been used to characterize “idiopathic” or “mysterious” diseases, which present phenotypes that may be due to genetic causes. The vast majority of such diseases are indeed “known” diseases that cannot be easily diagnosed by conventional candidate gene approaches, or require complicated differential diagnosis. For example, a recent study from the Baylor Whole Genome Laboratory reported a success rate of 25 percent to provide a genetic diagnosis for 250 patients, who presented a range of phenotypes suggesting potential genetic causes and ~80% were children with neurologic phenotypes [17]. An exome sequencing study on 100 patients with intellectual disability found that 53 of them carried *de novo* mutations, including 13 in known intellectual-disability genes, suggesting that exome sequencing is an effective diagnostic approach for diseases with extensive clinical heterogeneity [18]. Additionally, several studies have reported the discovery of completely novel diseases through genome/exome sequencing. One of the early examples was Ogden syndrome, a previously unreported infantile lethal disorder, involving a mutation in *NAA10* identified by us in 2011 using chromosome exon X capture and next-generation sequencing [19]. A more recent example is a novel genetic disease which we refer to as “Bookman syndrome”, a pediatric onset disease with neuromuscular and cardiac involvement and with clinical features similar to Glycogen Storage Disease Type IV. Although exome sequencing failed to identify the causes for the disease due to technical reasons, we applied genome and transcriptome sequencing and identified a disease-contributory mutation in *RBCK1* [20], which was further replicated by another group [21]. Besides finding causal genes for genetic diseases, genome/exome sequencing may also aid in the treatment of rare diseases. One of the earliest high-profile examples is the Pulitzer Prize-winning story of Nicholas Volker, a young boy who at the age of 17 months experienced perineal fistulae coupled with leakage of stool via holes spanning from the intestine to the skin [22]. Exome sequencing on this boy resulted in a diagnosis of an X-linked inhibitor of apoptosis deficiency. Based on this finding, an allogeneic hematopoietic progenitor cell transplant was performed on Volker. Altogether, these examples demonstrated the power of genome/exome sequencing in uncovering the genetic basis of idiopathic diseases and finding treatments for them, even when only a very small number of patient samples are available.

Among people with such diseases, those with idiopathic neuropathy present special challenges for genetic diagnosis [23,24]. Due to the heterogeneous nature of clinical presentation, the difficulty with characterizing phenotypes precisely, and the locus heterogeneity for the same diseases, many patients with inherited neuropathy may not obtain a genetic diagnosis by candidate gene testing. However, these patients may be more likely to receive a positive diagnosis by exome sequencing interrogating all genes in a somewhat unbiased manner, and multiple recent studies have demonstrated successful examples [25,26]. Frequently, after a candidate gene is identified, it is then found that an atypical phenotype accounts for earlier failed candidate gene approaches, so that genome/exome sequencing can indeed expand the clinical spectrum of previously reported or novel mutations in known disease genes, especially for patients with neuropathy [27]. Perhaps more importantly, genome/exome sequencing can nominate important candidate genes or candidate diseases, which guides the selection of functional assays to confirm the diagnosis of the disease.

**Figure 1:**
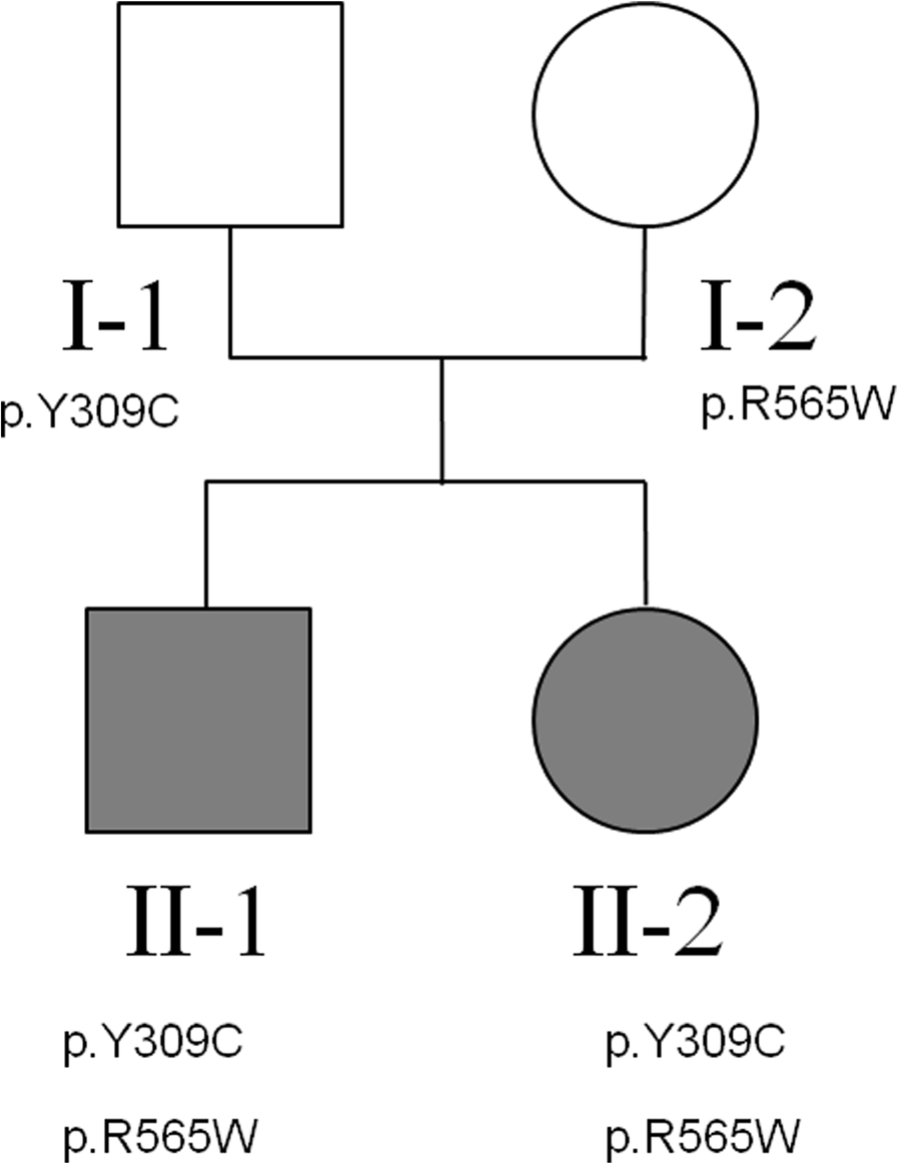
**Pedigree of the family quartet under study.** The carrier status for the causal mutations in *NAGLU* are marked below each subject in the family.

In the current study, we describe a case study where exome sequencing together with extensive biochemical tests pinpointed the disease-contributory gene for an idiopathic disease that failed to be diagnosed by conventional means. This study involved a Chinese family quartet (Figure 1) from Guangdong province, a relatively more developed area in China, but even top hospitals in these areas failed to give a diagnosis. It turned out that this is a known disease with a known genetic basis, but with an atypical phenotypic presentation such that appropriate biochemical tests were not performed until mutations pointing to this syndrome were identified. This ‘genotype-first’ approach has been discussed before, which suggests the need for the development of large, highly integrated networks of researchers, clinicians, and patient families, with the promise of improved therapies for subsets of patients [28,29]. Our study has strong implications for other extremely rare neuropathies, especially in areas where highly experienced medical geneticists may not be readily available.

## Methods

### Clinical description

The two probands in our study are siblings from a family in Guangzhou, Guangdong Province in China. The healthy parents are non-consanguineous, and had one affected son and one affected daughter (Figure 1). No evidence of any similar genetic disease was reported in family members. The study to identify disease genes through exome sequencing was approved by Jinan University First Affiliated Hospital Institutional Review Board (reference number: 2013–004). An informed consent for participation of study and publication of genetic results as well as individual medical details was obtained from each family member (or their guardians) who participated in the study.

**Figure 2:**
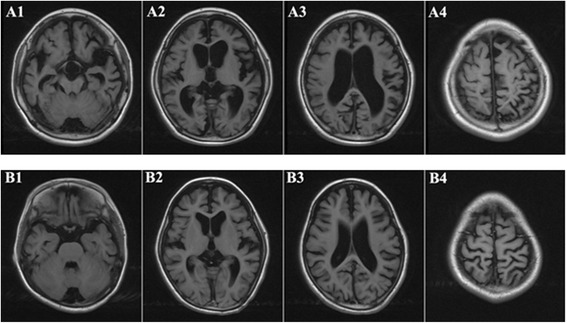
**Brain MRI of the two prodbands (A: brother 10 years old; B: sister 9 years old): Axial T1-weighted images showed that the hemisphere sulci and schizencephaly general broadened and deepened.** Double lateral and the third ventricle, cisterns were significantly dilated. The gray matter and white matter of the two hemisphere decreased. All of the above changes indicated ventricular dilatation and cortical atrophy in the two probands.

Individual II-1 is a 10 year old boy. He was born at term with normal birth parameters and good APGAR scores (9/10/10). The neonatal period was uneventful, and he had normal motor development during early childhood: he began to look up at 3 months, sit by himself at 5 months, stand up at 11 months, walk at 13 months, and speak at 17 months. He attended a regular kindergarten, without any signs of difference in intelligence, compared to his peers. Starting at age 6, the parents observed ever increasing behavioral disturbance for the boy, manifesting in multiple aspects of life. For example, he can no longer wear clothes by himself, cannot obey instruction from parents/teachers, can no longer hold subjects tightly in hand, which were all things that he could do before 6 years of age. In addition, he no longer liked to play with others; instead, he just preferred to stay by himself, and he sometimes fell down when he walked on the stairs, which had rarely happened at age 5. The proband continued to deteriorate: at age 9, he could not say a single word and had no action or response to any instruction given in clinical exams. Additionally, rough facial features were noted with a flat nasal bridge, a synophrys (unibrow), a long and smooth philtrum, thick lips and an enlarged mouth. He also had rib edge eversion, and it was also discovered that he was profoundly deaf and had completely lost the ability to speak. He also had loss of bladder control. The diagnosis of severe intellectual disability was made, based on Wechsler Intelligence Scale examination. Brain MRI demonstrated cortical atrophy with enlargement of the subarachnoid spaces and ventricular dilatation (Figure 2). Brainstem evoked potentials showed moderate abnormalities. Electroencephalography (EEG) showed abnormal sleep EEG.

Individual II-2 is a 9 years old girl. She was born at term, also with normal birth parameters. She began to stand at 11 months, walk with aid at 13 months, and speak at 17 months. At age 5, she was just like other children of similar age, with the ability to dress and sing, and count by herself. Starting at 6 years of age, she began to show regression of developmental patterns: she could not dress by herself anymore, and could not express even a single sentence or count numbers. Clinical examination revealed a coarse face with low anterior and posterior hairlines, prominent frontal bossing, thick eyebrows, synophrys (unibrow), hypertelorism, and thick lips. Growth parameters were normal. Her clinical course was also severe, with progressive neurodegeneration, behavioral problems (including hyperactivity, impulsivity, obstinacy, anxious behaviors and autistic-like behaviors), and hearing loss. The diagnosis of severe intellectual disability was made, based on Wechsler Intelligence Scale examination. Measuring activities of daily living showed extreme disability. Brain MRI demonstrated cortical atrophy with enlargement of the subarachnoid spaces and ventricular dilatation (Figure 2). Brainstem evoked potentials showed moderate abnormalities. EEG recording showed abnormal sleep EEG, just like her brother’s manifestation.

### Exome sequencing

We elected to perform exome sequencing on the probands, and to use parental samples for Sanger validation. Genomic DNA was extracted from peripheral blood for all family members by Qiagen DNeasy Blood & Tissue kit (Valencia, CA, USA). We obtained at least 5ug DNA from each sample, and all DNA samples passed quality control measures for exome sequencing.

Exome capture was carried out on 1 μg of genomic DNA, using the Agilent SureSelect^XT^ Human All Exon 50 Mb kit (Agilent Technologies, Santa Clara, CA, USA), which targets 50 Mb of coding exonic sequences, which were annotated by the GENCODE project, CCDS, miRbaseV14 and Sanger, as well as 10 base pairs of flanking sequence of each exonic region and non-coding RNA. The captured and amplified library was then loaded onto the Illumina Hiseq2000 sequencer. We generated 100 bp paired-end reads in the sequencing run, according to the manufacturer’s protocols. Raw images were processed by Illumina Pipeline v1.3.4 for base-calling with default parameters. Illumina SCS and CASAVA software were used for raw data processing and FASTQ file generation. In total, we obtained ~70 million reads per sample.

### Sequence alignment and variant calling

For bioinformatics analysis of the FASTQ data from exome sequencing, we used SeqMule (http://seqmule.usc.edu), which is an automated pipeline to execute multiple alignment algorithms and multiple variant calling software tools. Briefly, sequencing data was evaluated with FastQC (http://www.bioinformatics.babraham.ac.uk/projects/fastqc/). Short reads were aligned to reference genome (hg19) by BWA-MEM (version 0.7.4) [30] algorithm with default settings. Then we used three variant calling software tools, including the GATK version 3.1 [31], SAMtools version 0.1.19 [32] and FreeBayes version 0.9.14 [33] with default settings for exome sequencing. We selected the consensus variants using the “two of three algorithms” protocol, which specifies that variants that were detected by at least two algorithms will be present in the final consensus calling set.

### Variant annotation and prioritization

We used the ANNOVAR software [34] for functional annotation of variants and the Tute web application (tutegenomics.com) for identifying candidate variants and genes for the clinical phenotype. We used a “single-case analysis” pipeline to identify a list of candidate genes with the following criteria: (1) identify variants causing splicing or protein coding changes, including stop loss and stop gain variants; (2) remove variants with minor allele frequency (MAF) greater than 1% in the PopFreqMax database from ANNOVAR package [34], which include maximum allele frequency from the 1000 Genomes Project April 2012 release (five ethnicity groups), the NHLBI-6500 Exomes (two ethnicity groups), and the Complete Genomics 46 genomes (CG46) database; (3) imposed a recessive mode of inheritance, with at least two deleterious mutations found in each candidate genes in the proband, and with the mutations shared by both probands; (4) The resulting variants and genes were sorted based on relevance to user-supplied phenotypes, as well as annotations from clinical databases such as ClinVar (version 20140303) and HGMD (version 2014_2) [35].

### Validation by Sanger sequencing

Selected putative variants were examined among all family members using Sanger sequencing. Given the chromosomal position of variants, we designed PCR primers to amplify fragments harboring individual variants by Primer3 [36]. The PCR primers were designed to encompass the candidate position, ensuring that common SNPs are not covered by the primers. The ABI 3730 XL sequencer was used for sequencing, and the resulting *.AB1 files were loaded into the ABI Sequence Scanner Software v1.0 for further analysis and genotype calling. All sequence traces were manually reviewed to ensure the reliability of the genotype calls.

The primers used for chr17 :40695717(C > T) mutation are CCCGCCTCTTCCCCAACTC (forward) and GGACGCCTCCAGCCCTCAA (reverse). The primers used for chr17 :40693129 (A > G) mutation are AAACCAGGAGCTGTAGAGAAGT (forward) and CTGCCTACCCCTACTGACATCT (reverse).

### Structure modeling

We searched the Protein Data Bank (PDB) [37] for structure models of NAGLU. Although the structure for human NAGLU was not available, a previous study solved the crystal structure, catalytic mechanism, and inhibition of CpGH89 from Clostridium perfringens, a close bacterial homolog of NAGLU [38]. We downloaded the structure from X-ray diffraction with a resolution of 2.36 Å with binding of the ligand 2-Acetamindo-1,2-Dideoxynojirmycin (PDB identifier: 2VC9). We built a structure model for human NAGLU using I-TASSER [39], given high sequence identity. The template structure 2VC9 binds to 2-ACETAMIDO-1,2-DIDEOXYNOJIRMYCIN (PDB identifier: NOK). The position of NOK is decided by superpositioning our model to the template structure 2VC9 (shown in ball and stick). The 3D representation of the structure was generated by PYMOL (http://www.pymol.org/).

### Biochemical test in blood/urine

Enzyme activity was measured on peripheral blood leukocytes using fluorogenic substrate obtained from Moscerdam Substrates (Rotterdam, Netherlands); peripheral blood leukocytes was collected and sonicated to protein homogenates. Protein homogenates were incubated with Moscerdam substrate. The parameters for enzyme activity was obtained by measuring the fluorescence intensity and comparing with standard fluorescence intensity and concentration. The assays were performed according to the protocol at Department of Endocrinology and Metabolism, Guangzhou Women and Children’s Medical Center [40-44]. Urinary GAG was measured using dimethylmethylene blue/Tris by spectrophotometry and corrected for urinary creatinine (Cr) content [45,46]. The ratio of GAG/Cr (mg/mmol) was compared to age-matched normal controls (6 m-12 m, < 23.3; 12 m-36 m, <17.3; 3-5 years, <13.2; >5 years, < 9.7).

## Results

### Assessment of clinical information

The two probands were brother and sister one year apart in age, and both of them had perfectly normal development patterns in the first five years of age. Starting from age six, the probands suddenly began to develop progressive speech and cognitive decline with behavioral difficulties, motor function decline and hearing loss: they gradually lost the ability to talk, count, or walk. Rough facial features began to develop including synophrys (unibrow), thick lips and enlarged mouth. Brain MRI demonstrated cortical atrophy with enlargement of the subarachnoid spaces and ventricular dilatation (Figure 2). An extended list of clinical features is given in Materials and Methods. In the course of the clinical workup, multiple attempts for genetic diagnosis were made, including tests of chromosomal abnormalities and sequencing on a panel for metabolic diseases. Multiple types of blood screening tests and physical examinations were performed on the pedigree, especially the probands, to exclude the common inherited metabolic diseases and nervous system abnormalities. Additional factors were considered to exclude the possibility of food poisoning, environmental toxification and other non-genetic reasons. However, none of these attempts showed positive results, and these two probands were eventually referred to as having “mental deterioration of unknown origin”.

To research the causes of the diseases in this family, we examined all available phenotype information on the probands. Both parents are healthy and are non-consanguineous. No evidence of other genetic disease was reported in any additional family members. We suspected that this is an extremely rare or novel genetic disease, possibly following a recessive pattern of inheritance. Exome sequencing appeared to be the next natural choice to identify candidate mutations possibly contributing to this idiopathic disease.

### Exome sequencing identifies a prioritized list of candidate mutations

**Figure 3:**
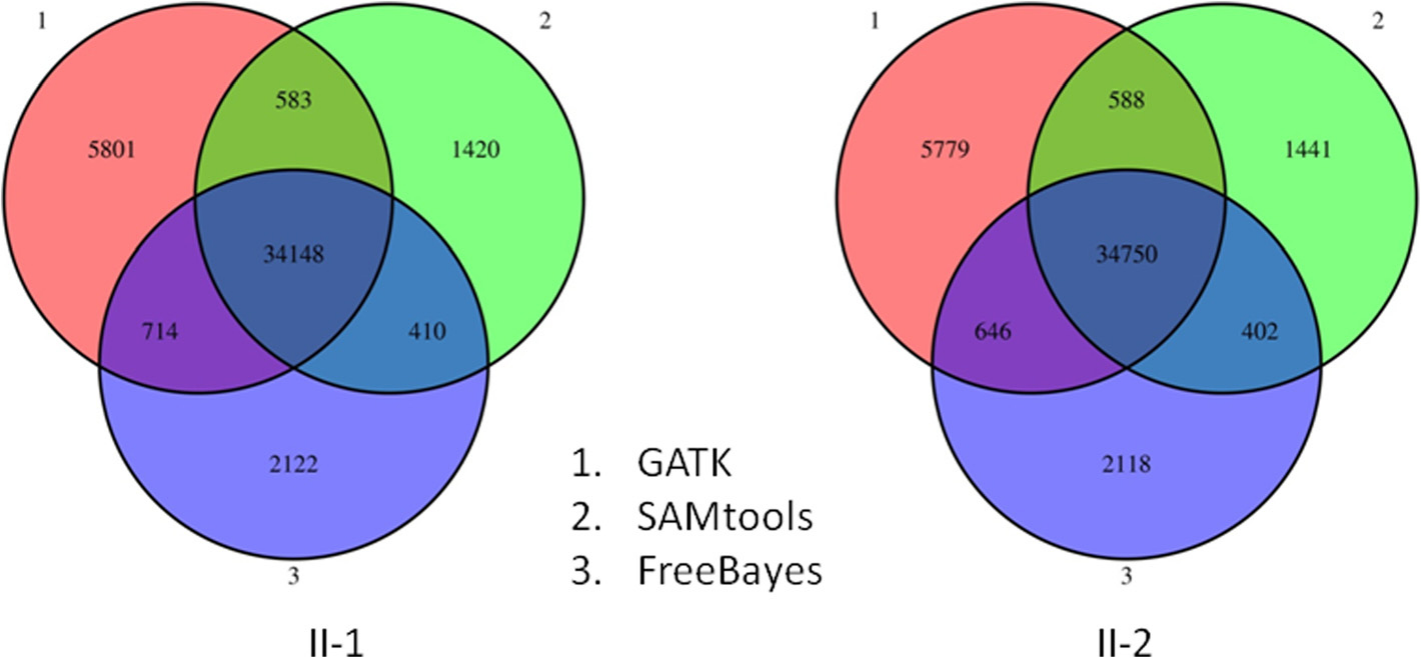
**Venn diagrams showing the overlap of different variant calling algorithms on two exomes (II-1 and II-2).** The three algorithms are (1) GATK, (2) SAMtools and (3) FreeBayes. The numbers denote the number of called variants.

To identify genetic causes for the disease in the family, we performed exome sequencing on the two probands with Agilent exon capture arrays, followed by sequencing on the Illumina HiSeq2000 platform. For each proband, we obtained approximately 70 million paired-end reads of 100 base pairs. The mapping rates for reads from brother (II-1) and sister (II-2) were 98.4% and 98.8%, respectively, and the on-target rates (fraction of reads mapped to designed exome regions) were 54.9% and 58.4%, respectively. We achieved a coverage of 68.2X and 71.3X over designed capture regions, and with 88.3% and 88.5% of target regions covered by ≥10 reads for brother and sister, respectively (82.0% and 82.8% and covered by ≥20 reads). Data analysis was performed using the SeqMule pipeline (http://seqmule.usc.edu), which is an automated pipeline for analysis of high-throughput sequencing data. It integrates multiple alignment algorithms and multiple variant calling software tools and gives user the flexibility to choose their preferred aligners and variant callers, and enables generation of consensus calls from multiple calling algorithms to improve reliability. We selected the consensus variants using the “two of three algorithms” protocol from GATK v3.1 [31], SAMtools v0.1.19 [32] and FreeBayes v0.9.14 [33], based on our previous work showing improved accuracy when using multiple calling algorithms [47]. The overlap of the three calling algorithms were presented in Figure 3, demonstrating substantial differences between the variant callers, even when the same alignment files were used. In total, 37,183 (2,268 indels) and 37,822 (2,312 indels) variants were identified from brother and sister respectively. To further identify likely causal genetic variants from exome data, we used the Tute web server which incorporates phenotype information, a variant scoring system and several clinical databases to rank variants and genes. *NAGLU* stands out as the most likely candidate gene for the disease under a recessive model (by requiring two predicted deleterious mutations in the same gene).

We identified two compound heterozygous mutations in *NAGLU* (N-acetylglucosaminidase, alpha), in both the probands (chr17:40695717C > T, c.1693C > T, p.R565W; chr17:40693129A > G, c.926A > G, p.Y309C). Neither variant was reported in the 1000 Genomes Project, the NHLBI-ESP6500 exome sequencing project or the Complete Genomics 46 genomes database. The first SNP was documented in dbSNP with identifier rs104894597. We evaluated the bioinformatics predictions on deleteriousness of non-synonymous variants from the dbNSFP database [48], and found that all algorithms gave largely consistent predictions that both are deleterious (Table 1), except that LRT predicted p.Y309C as “neutral”. *NAGLU* encodes a lysosomal enzyme that degrades heparan sulfate by hydrolysis of terminal N-acetyl-D-glucosamine residues in N-acetyl-alpha-D-glucosaminides, which is the fifth step of degradation of glycosaminoglycans (mucopolysaccharides). Defects in this gene are the cause of mucopolysaccharidosis type IIIB (MPS-IIIB), also known as Sanfilippo syndrome B [49]. This disease is characterized by the lysosomal accumulation and urinary excretion of heparan sulfate. The clinical severity of MPS-IIIB is highly variable, ranging from mild to severe, even in the same family, but typically includes progressive neurodegeneration, behavioral problems, mild skeletal changes, and shortened life span [50-52]. To date, over 119 unique disease contributory mutations underlying MPS IIIB have been identified in *NAGLU* in the Human Gene Mutation Database (HGMD) [53]. Both mutations in the probands were documented in HGMD as previously reported disease-contributory mutations [54,55]. Only one mutation (p.R565W) was documented in the ClinVar database (identifier: RCV000001633) as a “pathogenic” mutation.

**Table 1:** **Bioinformatics predictions of deleteriousness on the two non-synonymous variants in** ***NAGLU***

	**p.Y309C**	**p.R565W**
SIFT score	0	0
SIFT prediction	Deleterious	Deleterious
PolyPhen score	1	1
PolyPhen prediction	Probably damaging	Probably damaging
LRT score	0	0
LRT prediction	Neutral	Deleterious
Mutation Taster score	1	1
Mutation Taster prediction	Disease causing	Disease causing automatic
Mutation Assessor score	3.55	3.46
Mutation Assessor prediction	High	Medium
FATHMM score	−6.27	−5.82
FATHMM prediction	Deleterious	Deleterious
MetaSVM score	1.05	1.05
MetaSVM prediction	Deleterious	Deleterious
MetaLR score	0.99	0.98
MetaLR prediction	Deleterious	Deleterious
Tute score	0.92	0.92
Tute prediction	Deleterious	Deleterious

### Sanger validation of the candidate variants

**Figure 4:**
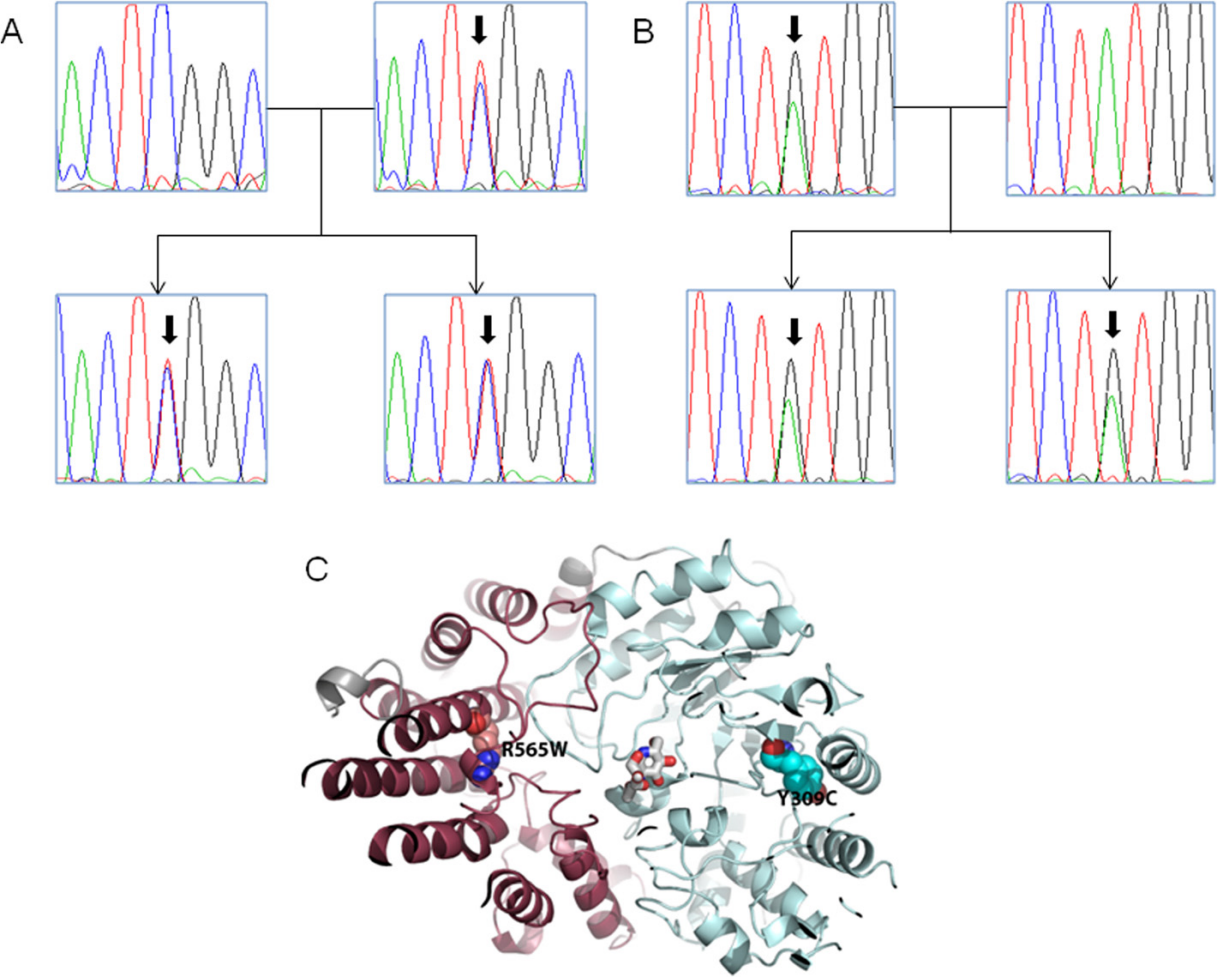
**Sanger validation and structural modeling of the two compound heterozygous candidate variants in** ***NAGLU*** **in the family quartet. (A)** Validation of the chr17 :40695717(C > T) mutation by Sanger sequencing in the family. **(B)** Validation of the chr17 :40693129 (A > G) mutation by Sanger sequencing in the family. **(C)** Structural location of mutation R565W and Y309C in *NAGLU*. We have built a structure model based on a close homolog structure (PDB identifier: 2VC9) of CpGH89 from *Clostridium perfringens*. The template structure 2VC9 binds to 2-ACETAMIDO-1,2-DIDEOXYNOJIRMYCIN (PDB identifier: NOK).

To validate the presence of the mutations and to identify whether they are recessively inherited, we performed Sanger sequencing on all the family members (Figure 4A,B). We found that the father and mother carried the p.Y309C and p.R565W and mutations, respectively, while the two probands inherited both variants from their parents, resulting in compound heterozygous mutations in *NAGLU.* Therefore, Sanger validation confirmed that these two mutations are inherited from different parents, which fits the known inheritance mode of the Sanfilippo syndrome.

### Functional impacts of the candidate variants by biochemical test

The analysis of candidate genes guided us to perform functional studies to evaluate whether MPS-IIIB or other types of MPS could explain the observed disease phenotypes in the family. We tested eight MPS related enzymes, including alpha-L-iduronidase, iduronate-2-sulfatase, heparan sulfate sulfatase, alpha N-acetylglucosaminidase, galactosamine 6-Sulfatase, β-galactosidase, arylsulfatase, β-glucuronidase, which are responsible for different subtypes of MPS (Table 2). The biochemical tests showed that alpha-N-acetylglucosaminidase, which is encoded by *NAGLU*, is completely inactive in both probands. Additionally, the level of alpha-L-iduronidase was slightly lower than reference value in one proband, but this could be due to variations in measurement. The biochemistry results were consistent with the bioinformatics analysis, and demonstrated that the two compound heterozygous mutations are indeed loss-of-function mutations.

**Table 2:** **Results of biochemical tests on the activity of MPS-related enzymes**

**Disease subtype**	**Enzyme**	**Gene**	**Value (sister)**	**Value (brother)**	**Reference value**	**Result**	Unit*
MPSI	alpha-L-iduronidase	IDUA	26.5	25.1	25.4-118.5	Borderline abnormal	nmol/mg/h
MPSII	iduronate-2-sulfatase	IDS	31.4	32.2	30-120	normal	nmol/mg/4 h
MPSIIIA	heparan sulfate sulfatase	SGSH	5.3	6.3	4.1-12	normal	nmol/mg/17 h
MPSIIIB	Alpha-N-acetyl-glucosaminidase	NAGLU	**0.0**	**0.0**	5-22	**abnormal**	nmol/mg/17 h
MPSIVA	galactosamine 6- sulfate Sulfatase	GAS	72.2	68.1	40-170	normal	nmol/mg/17 h
MPSIVB	β-galactosidase	lacZ	89.2	88.5	50.3-140.7	normal	nmol/mg/h
MPSVI	arylsulfatase	Ars	67.0	49.0	50.4-175.2	normal	nmol/mg/h
MPSVII	β-glucuronidase	GUSB	94.0	98.0	38.1-202.5	normal	nmol/mg/h

*concentrations of metabolites (nmol/mg) after hour of incubation.

To further validate the consequences of the lack of enzyme activity for alpha-N-acetylglucosaminidase, we performed urinary tests to measure the levels of metabolites for the enzyme (Table 3). The level of urinary glucosaminoglycan was extremely high in both probands, as opposed to an expected value of zero. Additionally, the substrates of the NAGLU enzyme, including dermatan sulfate and heparan sulfate, are both positive in urine, further suggesting that loss of function mutations in *NAGLU* leads to abnormal accumulation of sulfate in urine, which may explain the observed neuropathy.

**Table 3:** **Results from urinary test of glycosaminoglycan related parameters**

**Urinary test**	**Value (sister)**	**Value (brother)**	**Reference value**	**Result**	**Unit**
Urinary glycosaminoglycan (GAG)	528.1	413.8	0	abnormal	mg/L
Urinary glycosaminoglycan/Creatinine (GAG/Cr)	36.7	39.7	<8.4	abnormal	mg/mmol
Dermatan sulfate	**+**	**+**	**-**	abnormal	
Heparan sulfate	**+**	**+**	**-**	abnormal	
Keratan	-	-	-	normal	
Chondroitin sulfate	-	-	-	normal	

### Functional impacts of the candidate variants by structural modeling

To further understand the functional impacts of the two variants, we attempted to examine structure models for NAGLU. A previous study solved the crystal structure, catalytic mechanism, and inhibition of CpGH89 from Clostridium perfringens, a close bacterial homolog of *NAGLU* [38]. The structure was generated by X-ray diffraction with a resolution of 2.36 Å. We built a structure model for human NAGLU based on this template (PDB identifier: 2VC9) using I-TASSER [39], given the high sequence identity. The complete structure contains four domains (Figure 4C), including a N-terminal domain which is a putative family 32 carbohydrate-binding module (CBM) with the typical β-sandwich fold, a catalytic region comprises a small α/β domain, an elaborated (α/β)8 barrel, and an all α-helical domain that packs against the first three domains. We found that these two mutations were not directly within the active site (ligand-binding site) of *NAGLU*. Mutation Y309C is in the middle of the elaborated alpha/beta barrel (in blue) and R565W is located at the alpha-helical domain (in red) that packs against the binding site. Therefore, mutation Y309C may affect the structural stability of the alpha/beta barrel that constitutes the bottom of the active site. Mutation R565W may plays an important role in structural packing of the alpha-helical domain that contributes to the stability of the complete structure.

## Discussion

In this study, we present a case illustrating the power of NGS in clinical diagnosis of a rare disease in two siblings with somewhat atypical phenotypes, with a disease onset at ~ six years of age. Although this turned out to be a known disease with a known genetic cause, our experience in this study is nevertheless revealing in the context of a study conducted in China, where traditionally experienced medical geneticists are lacking. We suspected that had a more experienced medical geneticist reviewed the patient, biochemical tests on different types of MPS could have been ordered, and a diagnosis of MPS-IIIB may have been made without exome sequencing. However, in practice, numerous other tests were ordered and all turned out to be negative, to a point where the family was reluctant to pay for any additional tests. After exome sequencing, the mutation pointed us to a phenotype related to MPS, and therefore we performed biochemical tests in blood and urine to confirm this diagnosis. Therefore, exome sequencing in this particular context provided guidance for us to reach a final diagnosis, and this also illustrates the importance of supporting bioinformatics predictions with functional evidence.

Sanfilippo syndrome B (or MPS-IIIB) is an extremely rare disorder and the prevalence in China is unreported and unknown. Due to the rare nature of the disease, the incidence rate could vary significantly among different parts of the world. Using multiple ascertainment sources, one study obtained an incidence rate for Sanfilippo syndrome (form A + B + C) in western Australia for the period 1969 to 1996 of approximately 1 in 58,000 live births [56]. Since there were 5 people with type B in the total of 11 cases, this suggested an incidence of MPS-IIIB of approximately 1 in 127,600 live births. In different studies, an incidence of 1:24,000 was reported for all subtypes of MPS III [52]. A more recent study on the Australian population estimated an incidence rate of 1 in 200,000 [57]. Northern Ireland tends to have much lower incidence of MPS, and a report showed the rate of 1 in 280,000 for all subtypes of MPS III [58]. In the Netherland, the combined birth prevalence for all subtypes of MPS-III is 1.89 per 100,000 live births [59].

While we emphasize that the development of technologies has now enabled rapid and relatively cost-efficient identification of genetic causes in all parts of the world, the major bottleneck is still the identification of disease-contributory variants, which is heavily dependent on the bioinformatics analysis of raw data and functional interpretation of genetic variants. We used the SeqMule pipeline for variant calling, to take ‘2 out of 3’ consensus calls from three variant calling algorithms, in a way similar to what the 1000 Genomes Project has been implementing in their variant calling procedure. We also used ANNOVAR and the Tute web server for variant analysis focusing on missense, nonsense and splice variants. For the two missense variants in *NAGLU*, we used multiple prediction algorithms, including six from dbNSFP [48] (SIFT, PolyPhen, LRT, Mutation Taster, Mutation Assessor, FATHMM), two meta-scores built by us (MetaSVM, MetaLR), and the Tute score (tutegenomics.com), and found that these algorithms gave consistent predictions. However, the main reason we focused on *NAGLU* is that it is reported in clinical databases (ClinVar and HGMD) as containing mutations linked to disease. This fact underscores the importance of curated mutation databases in helping identify disease causal mutations from newly sequenced genomes or exomes.

## Conclusions

In conclusion, we have presented an example in China where exome sequencing preceded and guided the selection of functional validations, to lead to a diagnosis of a known disease with a somewhat atypical presentation. With the ever decreasing cost of NGS and the rapid adoption of NGS in clinical settings, we expect that genome/exome sequencing will be increasingly useful in the diagnosis of rare genetic disorders with atypical phenotypic presentations, including in areas of the world where experienced medical geneticists are scarce.
